# Three Doses of Hepatitis B Vaccine or Four Doses in Chronic Renal Failure

**DOI:** 10.5812/hepatmon.10040

**Published:** 2013-03-14

**Authors:** Zahra Khazaeipour, Farokhlagha Ahmadi

**Affiliations:** 1 Brain and Spinal Injury Research Center, Neuroscience Institute, Tehran University of Medical Sciences, Tehran, IR Iran; 2 Nephrology Research Center, Imam Khomeini Hospital Complex, Tehran University of Medical Sciences, Tehran, IR Iran

**Keywords:** Hepatitis B, Vaccination, Kidney Failure, Chronic

Dear Editor,

Regarding the letter to our article (1) entitled “Do We Have to Shift Three Doses of Hepatitis B Vaccine Instead of Four doses in Chronic Renal Failure: Think before Action ” (2), it should be considered that we hypothesized “four doses of 40 μg may be better than three doses of 20 μg Hepatitis B Vaccine, in Chronic Renal Failure”. We had two kinds of comparison; the comparison between two groups of study and the comparison between four doses and three doses in 40 μg group. In comparison between two groups, the mean antibody titers after four doses of 40 μg (182.2 ± 286.7 mIU/ml), was higher than three doses of 20 μg vaccine (107.6 ± 192.1 mIU/ml), this result was not significant. This may be due to small sample size, and large standard deviation of HBs Ab levels. In comparison between 4 doses and 3 doses in 40 μg group, the seroconversion rate after 4 doses of 40 μg (21/26, 80.8%), was higher than that after three doses of 40 μg (20/26, 77%). The mean HBs Ab level after 4 doses of 40 μg (182.2 ± 286.7) was significantly higher than that attained after 3 doses of the 40 μg vaccine (96.9 ± 192.1) (P = 0.004). About the sample size: The sample size that we calculated was 137 patients, but in the period of the study all the available patients who met the inclusion criteria were 64 patients. This was our limitation about sample size that we have mentioned in discussion. At the beginning of results we explained about the number of patients: “Of 64 predialysis patients, two patients died, two required dialysis, and 9 were eliminated from the study because of problems reaching Tehran from other cities. There were no significant differences in the frequency and etiology of withdrawal between the two groups.” So we had 51 patients completed the 8-month follow-up (26 patients received 4 doses of 40 μg, and 25 patients 3 doses of 20 μg). The glomerular filtration rate (GFR) was calculated by Cockcroft-Gault formula that gives the best estimation of GFR, and the performance is close to that of Modification of Diet in Renal Disease (MDRD) equations (3). In conclusion we still use the method of three doses of 20 μg Hepatitis B Vaccine, in Chronic Renal Failure, because we didn’t find strong reason to change this vaccination method.
